# Role of Imprinting Disorders in Short Children Born SGA and Silver-Russell Syndrome Spectrum

**DOI:** 10.1210/clinem/dgaa856

**Published:** 2020-11-24

**Authors:** Tomoko Fuke, Akie Nakamura, Takanobu Inoue, Sayaka Kawashima, Kaori Isono Hara, Keiko Matsubara, Shinichiro Sano, Kazuki Yamazawa, Maki Fukami, Tsutomu Ogata, Masayo Kagami

**Affiliations:** 1 Department of Molecular Endocrinology, National Research Institute for Child Health and Development, Tokyo, Japan; 2 Department of Pediatrics, Hokkaido University Graduate School of Medicine, Sapporo, Japan; 3 Department of Pediatrics, Hamamatsu University School of Medicine, Hamamatsu, Shizuoka, Japan; 4 Medical Genetics Center, National Hospital Organization Tokyo Medical Center, Tokyo, Japan

**Keywords:** SGA, short stature, Silver-Russell syndrome, imprinting disorder

## Abstract

**Background:**

(Epi)genetic disorders associated with small-for-gestational-age with short stature (SGA-SS) include imprinting disorders (IDs). Silver-Russell syndrome (SRS) is a representative ID in SGA-SS and has heterogenous (epi)genetic causes.

**Subjects and Methods:**

To clarify the contribution of IDs to SGA-SS and the molecular and phenotypic spectrum of SRS, we recruited 269 patients with SGA-SS, consisting of 103 and 166 patients referred to us for genetic testing for SGA-SS and SRS, respectively. After excluding 20 patients with structural abnormalities detected by comparative genomic hybridization analysis using catalog array, 249 patients were classified into 3 subgroups based on the Netchine-Harbison clinical scoring system (NH-CSS), SRS diagnostic criteria. We screened various IDs by methylation analysis for differentially methylated regions (DMRs) related to known IDs. We also performed clinical analysis.

**Results:**

These 249 patients with SGA-SS were classified into the “SRS-compatible group” (n = 148), the “non-SRS with normocephaly or relative macrocephaly at birth group” (non-SRS group) (n = 94), or the “non-SRS with relative microcephaly at birth group” (non-SRS with microcephaly group) (n = 7). The 44.6% of patients in the “SRS-compatible group,” 21.3% of patients in the “non-SRS group,” and 14.3% in the “non-SRS with microcephaly group” had various IDs. Loss of methylation of the *H19*/*IGF2*:intergenic-DMR and uniparental disomy chromosome 7, being major genetic causes of SRS, was detected in 30.4% of patients in the “SRS-compatible group” and in 13.8% of patients in the “non-SRS group.”

**Conclusion:**

We clarified the contribution of IDs as (epi)genetic causes of SGA-SS and the molecular and phenotypic spectrum of SRS. Various IDs constitute underlying factors for SGA-SS, including SRS.

Approximately 10% of babies born small-for-gestational-age (SGA), a condition for babies with birth weight (BW) and/or birth length (BL) less than those expected for the gestational age and sex, do not have catch-up growth until 2 years of age (1, 2). These children are categorized as short children born SGA (SGA-SS). SGA is mainly defined by the following criteria: (i) below the 10th percentile for both BW and BL (ICD10, ICD10data.com), and (ii) below −2 standard deviation score (SDS) for BW and/or BL for the gestational age and sex of the population (3-5). The causes of SGA are multifactorial, including environmental, maternal, paternal, placental, and fetal factors (1). Among them, multiple (epi)genetic disorders, which are considered as fetal factors, could lead to SGA-SS.

(Epi)genetic disorders causing SGA-SS include genetic syndromes caused by defects in genes associated with human growth, imprinting disorders (IDs), and pathogenic copy number variants (PCNVs). Among them, IDs are caused by abnormal gene expression of the imprinted genes. Imprinted genes are expressed in a parental-origin-specific manner and are associated with fetal and placental growth (6). Parental-origin-specific gene expression is regulated by imprinting control regions in differentially methylated regions (DMRs) with parental-origin-specific DNA methylation patterns (6). SGA-SS is frequently observed in patients with IDs, such as Silver-Russell syndrome (SRS), Temple syndrome (TS14), Prader-Willi syndrome (PWS), maternal uniparental disomy chromosome 16 (UPD(16)mat), and maternal uniparental disomy chromosome 20 (UPD(20)mat) (7-11).

SRS is a representative ID associated with SGA-SS, and the diagnosis of SRS is based on clinical diagnostic criteria. The Netchine-Harbison clinical scoring system (NH-CSS) is the clinical diagnostic criteria for SRS having the most sensitive and the highest negative predictive value (12). A consensus statement recommends using NH-CSS as the clinical diagnostic criteria for SRS (13). Major genetic causes of SRS are loss of methylation of the *H19*/*IGF2*:intergenic (IG)-DMR (*H19*LOM) and maternal uniparental disomy chromosome 7 (UPD(7)mat) (7, 13); however, some patients with *H19*LOM and UPD(7)mat did not satisfy NH-CSS criteria, and were diagnosed as SGA-SS (12). Moreover, some patients with etiologies other than *H19*LOM and UPD(7)mat met NH-CSS criteria and were diagnosed as SRS (12, 14).

Previous work has examined the contribution of IDs to SGA-SS; however, the number of patients involved was not so large, and clinical assessment using clinical diagnostic criteria for SRS was not performed (15). To clarify the contribution of IDs to SGA-SS and the spectrum of molecular and phenotypic SRS, we carried out molecular and clinical analyses for 249 patients with SGA-SS. These patients were referred to us for genetic testing for SRS and SGA-SS. The following is a large comprehensive study investigating the etiologies related to IDs and the clinical features in patients with SGA-SS who were evaluated based on NH-CSS.

## Patients and Methods

### Ethics approval and consent to participate

This study was approved by the Institutional Review Board Committee at the National Center for Child Health and Development and performed after obtaining written informed consent from patients or patients’ parents to participate in this study and for publication of the clinical and molecular information.

### Patients

**Figure 1.**
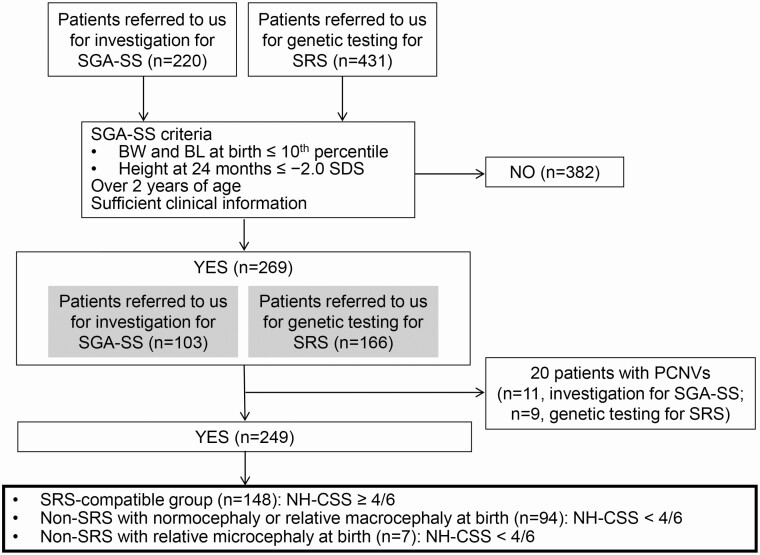
Inclusion criteria for this study. Abbreviations: BL, birth length; BW, birth weight; NH-CSS, Netchine-Harbison clinical scoring system; PCNV, pathogenic copy number variants; SDS, standard deviation score; SGA-SS, short children born small-for-gestational-age; SRS, Silver-Russell syndrome.

From 2002 to 2019 we recruited 220 patients referred to us for investigation for SGA-SS and 431 patients referred to us for genetic testing for SRS (Fig. 1). Among them, 269 patients met inclusion criteria (Fig. 1). Subsequently, we excluded 20 patients with structural abnormalities detected by array comparative genomic hybridization (aCGH) using catalog array. Finally, we screened various IDs in 249 patients with SGA-SS, including 92 patients referred to us for investigation for SGA-SS and 157 patients referred to us for genetic testing for SRS. Clinical features of patients were evaluated by the attending physicians, consisting of general pediatricians, neonatologists, pediatric endocrinologists, and pediatric geneticists. We collected the clinical information from each attending physician using a questionnaire. When we required more clinical data related to NH-CSS and/or specific etiology in some patients, we contacted their attending physicians again. Inclusion criteria for this study are shown in Fig. 1. We assessed their BW, BL, and height at 24 months for evaluating whether they had SGA-SS. If we could not obtain height at 24 months, we evaluated the height at the closest age over 24 months before initiating growth hormone treatment. We included patients in this study with both their BW and BL less than the 10th percentile of those expected for the gestational age and sex and their height at 24 months less than −2 SDS. We classified these patients into 3 subgroups based on NH-CSS consisting of 6 key items, as follows: (i) SGA, BW and/or BL are ≤ −2 SDS for gestational age; (ii) postnatal growth failure, height at 24 ± 1 months ≤ −2 SDS or height ≤ −2 SDS below mid-parental target height; (iii) relative macrocephaly at birth, ie, SDS for BW or BL minus SDS for birth occipitofrontal circumference (BOFC) ≤ −1.5; (iv) prominent forehead during early childhood; (v) body asymmetry; and (vi) feeding difficulties (current use of a feeding tube or cyproheptadine for appetite stimulation) during early childhood and/or low body mass index (BMI) ≤ −2 SDS at 24 months. The 249 patients with SGA-SS were classified into 3 subgroups. Patients with 4 or more NH-CSS items were classified into the “SRS-compatible group.” Patients with less than 4 NH-CSS items with normocephaly or relative macrocephaly at birth were classified into the “non-SRS with normocephaly or relative macrocephaly at birth group (non-SRS group),” and patients with less than 4 NH-CSS items with relative microcephaly at birth were classified into the “non-SRS with relative microcephaly at birth group (non-SRS with microcephaly group).” Based on the previous study, relative microcephaly was defined as the following: head circumference SDS below height SDS and weight SDS at birth (13).

### Molecular analysis

Genomic DNA of patients was extracted from leukocytes of peripheral blood. First, we performed aCGH analysis using a 60K catalog array (catalog number G4827A, Agilent Technologies, Palo Alto, CA) to detect PCNVs. To access the PCNVs, we found copy number variants (CNV) that had 3 or more continuous probes with aberrant signals. Then we evaluated whether these CNVs were pathogenic or not by referring to the Database of Genomic Variants (http://dgv.tcag.ca/dgv/app/home) and previous reports. Eventually, when CNVs were detected in our study involved in the regions causing known microdeletion or microduplication syndromes, we defined those CNVs as PCNVs. Next, to detect IDs, we performed methylation analysis with pyrosequencing for 9 DMRs related to known IDs, namely, the *H19*/*IGF2*:IG-DMR on chromosome 11, *PEG10*:transcription start site (TSS)-DMR and *MEST*:alt-TSS-DMR on chromosome 7, *PLAGL1*:alt-TSS-DMR on chromosome 6, *KCNQ1OT1*:TSS-DMR chromosome 11, *MEG3*/*DLK1*:IG-DMR and *MEG3*:TSS-DMR on chromosome 14, *SNURF:*TSS-DMR on chromosome 15, and *GNAS*-*A*/*B*:TSS-DMR on chromosome 20, as previously reported (7, 8). Furthermore, to detect UPD(16)mat, we performed methylation analysis for the *ZNF597*:TSS-DMR on chromosome 16 (10). Patients with hypomethylation of the *H19*/*IGF2*:IG-DMR were diagnosed as *H19*LOM. For patients with hypermethylation of both the *PEG10*:TSS-DMR and *MEST*:alt-TSS-DMR, we performed microsatellite analysis for chromosome 7 in patients and their parents, and confirmed UPD(7)mat (7, 16). For patients with the abnormal methylation levels of the DMR(s) related to IDs other than *H19*LOM and UPD(7)mat, such as TS14, PWS, UPD(6)mat, UPD(16)mat, and UPD(20)mat, we conducted structural analysis to detect micro-deletions and/or duplications involving the imprinted regions. For structural analysis, we used methylation-specific multiplex ligation-dependent probe amplification (MS-MLPA, MRC Holland, Amsterdam, Netherlands) analysis for each imprinted region (ME030 for 11p15.5, ME028 for 15q11.3–5, ME031 for 20q13) and/or aCGH analysis using custom built array (Design ID 080262, Agilent Technologies, Palo Alto, CA) and/or genome-wide comparative genomic hybridization and single-nucleotide polymorphism array (catalog number G4890A, Agilent Technologies, Palo Alto, CA), according to the manufacturer’s instructions. For patients with abnormal methylation levels of the DMRs related to known IDs other than *H19*LOM and UPD(7)mat, but not structural abnormalities, we carried out microsatellite analysis for chromosomes 6 (16), 14 (8), 15 (17), 16 (10), and 20 (11) using patients’ and their parental genomic DNA samples. If patients had aberrant methylated DMR(s) other than their disease-related DMR(s), we considered these patients as having multilocus imprinting disturbance (MLID).

## Results

### Molecular analysis

**Figure 2.**
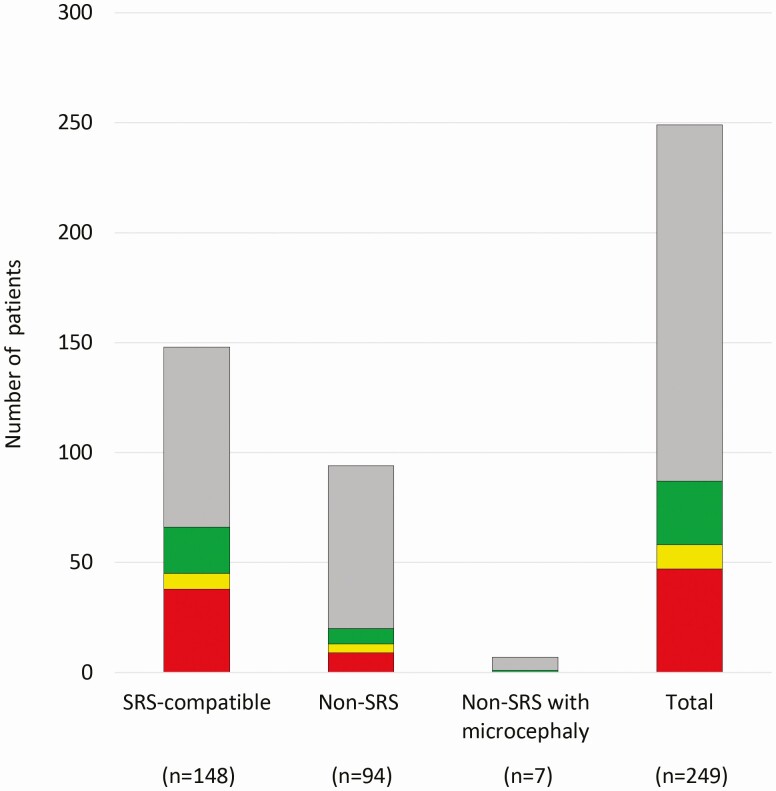
The frequency of etiologies in each subgroup. The red, yellow, green, and gray rectangles represent the frequency of *H19*LOM, UPD(7)mat, imprinting disorders other than *H19*LOM and UPD(7)mat, and unknown etiologies. *H19*LOM, loss of methylation of the *H19*/*IGF2*:intergenic differentially methylated region; UPD(7)mat, uniparental disomy chromosome 7.

The results of molecular analyses are summarized in Fig. 2 (see also Table 1). Of 249 patients with SGA-SS, *H19*LOM and UPD(7)mat, which are major genetic causes of SRS, were detected in 58 patients (23.3%), and IDs other than *H19*LOM and UPD(7)mat were in 29 patients (11.6%). Twenty patients with IDs other than *H19*LOM and UPD(7)mat were previously reported (see Table 1 and Table 2) (8, 10, 11, 18-20). These 249 patients with SGA-SS were classified into the SRS-compatible group (n = 148), non-SRS group (n = 94), and non-SRS with microcephaly group (n = 7). *H19*LOM and UPD(7)mat were identified in 30.4%, 13.8%, and none of patients in the SRS-compatible group, the non-SRS group, and the non-SRS with microcephaly group, respectively. IDs other than *H19*LOM and UPD(7)mat were identified in 14.2% of patients in the SRS-compatible group, in 7.4% of patients in the non-SRS group, and in 14.3% of patients in the non-SRS with microcephaly group. Particularly, 8 patients with TS14 were included in the SRS-compatible group. The non-SRS with microcephaly group had the lowest detection rate (14.3%) for IDs in patients.

**Table 1.** Imprinting Disorders Detected in This Study

	SRS-compatible	Non-SRS	Non-SRS with microcephaly	Total	
	n = 148	n = 94	n = 7	n = 249	
**Genetic causes of SRS**	45 (30.4%)	13 (13.8%)	0		
*H19*LOM	38	9	0	47	58/249 (23.3%)
UPD(7)mat	7	4	0	11	
**Imprinting disorders other than *H19*LOM and UPD(7)mat**	21 (14.2%)	7 (7.4%)	1 (14.3%)		
Temple syndrome (8, 18)	8	3		11	29/249 (11.6%)
UPD(20)mat (11)	4	1		5	
UPD(6)mat	1	2		3	
Prader-Willi syndrome	2		1	3	
11p15 maternal duplication (19)	2	1		3	
UPD(16)mat (10)	2			2	
Parthenogenesis	1			1	
UPD(11)mat mosaic (20)	1			1	
**Unknown**	82 (55.4%)	74 (78.7%)	6 (85.7%)		162/249 (65.1%)

Abbreviations: *H19*LOM, loss of methylation of the *H19*/*IGF2*:intergenic differentially methylated region; SRS, Silver-Russell syndrome; UPD(6)mat, maternal uniparental disomy chromosome 6; UPD(7)mat, maternal uniparental disomy chromosome 7; UPD(11)mat, maternal uniparental disomy chromosome 11; UPD(16)mat, maternal uniparental disomy chromosome 16; UPD(20)mat, maternal uniparental disomy chromosome 20.

**Table 2.** Clinical Features of the Patients With Imprinting Disorders Other Than H19LOM and UPD(7)mat

	TS14	UPD(20)mat	UPD(6)mat	PWS	11p15 maternal duplication	UPD(16)mat	UPD(11) mat mosaic	Partheno-genesis	Total
	n = 11	n = 5	n = 3	n = 3	n = 3	n = 2	n = 1	n = 1	
	(UPD = 6, Epi = 5)			(UPD = 1, UPD or Epi = 2)					
Subgroup based on NH-CSS									
SRS-compatible	8	4	1	2	2	2	1	1	21/29 (72.4%)
Non-SRS	3	1	2	0	1	0	0	0	7/29 (24.1%)
Non-SRS with microcephaly ^*a*^	0	0	0	1	0	0	0	0	1/29 (3.4%)
BOFC in (SDS) median (minimum to maximum) ^*a*^	−1.75 (−3.28 to −0.04)	−1.46 (−1.93 to −1.08)	−1.63 (−1.91 to −0.87)	−3.31 (−4.10 to −1.18)	−1.85 (−2.19 to −1.02)	−0.93 (−1.02 to −0.84)	–	–	
Number of cases with relative microcephaly ^*a*^	1	0	0	1	0	0	–	–	
NH-CSS items									
BW and/or BL ≤ −2 SDS ^*a*^	11/11 (100%)	4/5 (80.0%)	3/3 (100%)	3/3 (100%)	3/3 (100%)	2/2 (100%)	+	+	28/29 (96.6%)
Postnatal growth failure ^*b*, *c*, *d*^	11/11 (100%)	5/5 (100%)	3/3 (100%)	3/3 (100%)	3/3 (100%)	2/2 (100%)	+	+	29/29 (100%)
Relative macrocephaly at birth ^*e*^	6/11 (54.5%)	3/5 (60.0%)	3/3 (100%)	1/2 (50.0%)	3/3 (100%)	1/2 (50.0%)	+	+	19/28 (67.9%)
Protruding forehead	7/11 (63.6%)	3/5 (60.0%)	1/3 (33.3%)	2/3 (66.7%)	2/3 (66.7%)	2/2 (100%)	+	+	19/29 (65.5%)
Body asymmetry	3/11 (27.3%)	0/5 (0%)	0/3 (0%)	2/3 (66.7%)	0/3 (0%)	1/2 (50.0%)	+	+	8/29 (27.6%)
Feeding difficulties and/or low BMI ^*b*^	6/11 (54.5%)	5/5 (100%)	1/3 (33.3%)	3/3 (100%)	2/3 (66.7%)	2/2 (100%)	+	+	21/29 (72.4%)
Triangular face	6/11 (54.5%)	4/5 (80.0%)	2/3 (66.7%)	1/3 (33.3%)	2/3 (66.7%)	2/2 (100%)	–	+	18/29 (62.1%)
Fifth finger clinodactyly	8/11 (72.7%)	1/5 (20.0%)	1/3 (33.3%)	0/3 (0%)	0/3 (0%)	1/2 (50.0%)	+	+	13/29 (44.8%)
Muscular hypotonia	5/11 (45.5%)	2/5 (40.0%)	0/3 (0%)	3/3 (100%)	1/2 (50.0%)	1/2 (50.0%)	–	–	12/28 (42.9%)
Speech delay	5/11 (45.5%)	3/5 (60.0%)	0/3 (0%)	3/3 (100%)	3/3 (100%)	1/2 (50.0%)	+	+	17/29 (58.6%)
Congenital heart disease	0/9 (0%)	0/3 (0%)	0/3 (0%)	3/3 (100%)	0/3 (0%)	1/2 (50.0%)	–	–	4/25 (16.0%)
Genitourinary anomaly	0/9 (0%)	0/5 (0%)	2/3 (66.7%)	3/3 (100%)	0/3 (0%)	1/2 (50.0%)	+	–	7/27 (25.9%)
References	8, 18	11			19	10	20		

Abbreviations: BL, birth length; BMI, body mass index; BOFC, birth occipitofrontal circumference; BW, birth weight; Epi, epimutation; *H19*LOM, loss of methylation of the *H19*/*IGF2*: intergenic differentially methylated region; NH-CSS, Netchine-Harbison clinical scoring system; PWS, Prader-Willi syndrome; SDS, standard deviation score; SRS, Silver-Russell syndrome; TS14, Temple syndrome; UPD, uniparental disomy; UPD(6)mat, maternal uniparental disomy chromosome 6; UPD(7)mat, maternal uniparental disomy chromosome 7; UPD(11)mat, maternal uniparental disomy chromosome 11; UPD(16)mat, maternal uniparental disomy chromosome 16; UPD(20)mat, maternal uniparental disomy chromosome 20.

^*a*^ Birth weight, length, and head circumference were evaluated by sex-matched and gestational-age-matched Japanese reference data (http://jspe.umin.jp/medical/chart_dl.html). We adopted “head circumference SDS below height and weight SDS” as the definition of relative microcephaly based on the consensus statement (13).

^*b*^ Postnatal height, BMI, and weight were evaluated by sex-matched and age-matched Japanese reference data (http://jspe.umin.jp/medical/chart_dl.html).

^*c*^ If we did not get information at 24 ± 1 months, we used the data at the nearest measure available older than 25 months before initiating growth hormone treatment.

^*d*^ Height at 24 ± 1 months ≤ −2 SDS or height ≤ −2 SDS below mid-parental target height. Mid-parental target height was calculated as follows: ((father’s height + mother’s height)/2) + 6.5 cm for boys and −6.5 cm for girls.

^*e*^ Head circumference at birth ≥ 1.5 SDS above birth length and/or weight SDS. Head circumference at birth was evaluated by sex-matched and gestational-age–matched Japanese reference data (http://jspe.umin.jp/medical/chart_dl.html).

Three patients (3.4%) of 87 patients with various IDs had MLID. Two patients showed obvious *H19*LOM and 1 patient had extremely low methylation levels of the *MEG3*:TSS-DMR. Patient 1 with MLID (MLID 1) showed mild hypomethylation of the *MEG3*/*DLK1*:IG-DMR and *MEG3*:TSS-DMR besides *H19L*OM. Patient 2 with MLID (MLID 2) showed mild hypomethylation of the *GNAS*-*A*/*B*:TSS-DMR besides *H19L*OM. Patient 3 with MLID (MLID 3) showed mild *H19L*OM besides hypomethylation of the *MEG3*:TSS-DMR, as previously reported (18). A single patient showed abnormal methylation levels in all examined DMRs due to parthenogenesis.

### Clinical analysis

#### Clinical characteristics for each etiology

Clinical data for each subgroup in patients with *H19*LOM, UPD(7)mat, IDs other than *H19*LOM and UPD(7)mat, and unknown etiology are summarized in Table 3. A total of 157 patients out of 249 patients were suspected to have SRS, and the remaining patients were diagnosed with SGA-SS (Fig. 1, Table 3). Fifty-two patients with *H19*LOM and UPD(7)mat, and 20 patients with IDs other than *H19*LOM and UPD(7)mat had clinically suspected SRS. Clinical manifestations in patients with each etiology are summarized in Table 3. Phenotypic comparison between *H19*LOM and UPD(7)mat showed that patients with *H19*LOM had typical SRS phenotypes, such as severe prenatal and postnatal growth failure and higher frequency of the remaining clinical features in NH-CSS. BOFC of patients with *H19*LOM were more preserved than those of patients with other etiologies. Patients with UPD(7)mat showed less serious prenatal growth failure compared to patients with *H19*LOM, showed severe postnatal growth failure similar to patients with *H19*LOM, and had a high frequency of relative macrocephaly at birth (Table 3). Patients with IDs other than *H19*LOM and UPD(7)mat showed feeding difficulties and/or low BMI in over 70%. Of clinical features frequently observed in SRS patients, triangular face was observed in approximately 90% of patients with *H19*LOM and in 70% of patients with UPD(7)mat. Fifth finger clinodactyly was detected in more than 70% of patients with *H19*LOM and in about 45% of patients with IDs other than *H19*LOM and UPD(7)mat. Muscular hypotonia was observed at relatively high frequency in patients with IDs other than *H19*LOM and UPD(7)mat, compared to those with other etiologies. Speech delay was detected in 40% and 58.6% of patients with UPD(7)mat and IDs other than *H19*LOM and UPD(7)mat, respectively. Congenital heart diseases (CHDs) were observed in 16% of patients with IDs other than *H19*LOM and UPD(7)mat. Genitourinary anomalies were observed in approximately 30% of patients with *H19*LOM.

**Table 3.** Clinical Manifestations in Patients With Each Etiology

Etiologies	*H19*LOM			UPD(7)mat			IDs other than *H19*LOM and UPD(7)mat			Unknown		
Number of patients with each etiology	47			11			29			162		
Subgroups based on NH-CSS	SRS-compatible	Non-SRS	Non-SRS with microcephaly	SRS-compatible	Non-SRS	Non-SRS with microcephaly	SRS-compatible	Non-SRS	Non-SRS with microcephaly	SRS-compatible	Non-SRS	Non-SRS with microcephaly
Number of patients in each subgroup	38 (80.9%)	9 (19.1%)	0 (0%)	7 (63.6%)	4 (36.4%)	0 (0%)	21 (72.4%)	7 (24.1%)	1 (3.4%)	82 (50.6%)	74 (45.7%)	6 (3.7%)
Clinical diagnosis SGA-SS	2	2	0	2	0	0	3	5	1	21	51	5
Clinical diagnosis SRS	36	7	0	5	4	0	18	2	0	61	23	1
BW and/or BL ≤ –2 SDS^*a*^	38/38	8/9	0	7/7	4/4	0	20/21	7/7	1/1	82/82	72/74	5/6
BW (SDS) median (minimum to maximum)^*a*^	–3.73 (–7.60 to –1.80)			–3.06 (–4.33 to –2.00)			–2.99 (–5.35 to –1.86)			–2.79 (–5.31 to –1.36)		
BW (SDS) median (minimum to maximum)^*a*^	–3.77 (–7.60 to –1.80)	–2.85 (–4.32 to –1.86)	–	–3.14 (–4.33 to –2.00)	–2.63 (–3.38 to –2.07)	–	–2.80 (–5.35 to –1.86)	–3.20 (–4.38 to –2.13)	–3.70	–3.23 (–4.95 to –1.73)	–2.49 (–5.31 to –1.36)	–2.42 (–4.72 to –1.70)
BL in (SDS) median (minimum to maximum)^*a*^	–3.27 (–5.94 to –1.41)			–2.98 (–4.30 to –1.47)			–2.53 (–4.26 to 0.98)			–2.45 (–4.60 to –1.28)		
BL in (SDS) median (minimum to maximum)^*a*^	–3.35 (–5.94 to –2.00)	–2.55 (–5.12 to –1.41)	–	–2.98 (–3.50 to –1.57)	–2.45 (–4.30 to –1.47)	–	–2.66 (–4.26 to –1.41)	–2.38 (–4.21 to 0.98)	–2.51	–2.85 (–4.59 to –1.36)	–2.20 (–4.60 to –1.28)	–2.25 (–2.78 to –1.90)
BOFC in (SDS) median (minimum to maximum)^*a*^	–0.88 (–2.79 to 1.65)			–1.31 (–2.26 to 0.35)			–1.68 (–4.10 to –0.04)			–1.48 (–5.48 to 9.96)		
BOFC in (SDS) median (minimum to maximum)^*a*^	–0.54 (–2.79 to 1.65)	–1.66 (–2.06 to 0.51)	–	–1.27 (–2.26 to 0.35)	–1.31 (–1.59 to –0.22)	–	–1.65 (–3.31 to –0.04)	–1.63 (–2.50 to –0.87)	–4.10	–1.48 (–4.92 to 9.96)	–1.34 (–3.61 to 1.09)	–3.15 (–5.48 to –2.34)
Postnatal growth failure^*b*,*c*,*d*^	38/38	9/9	0	7/7	4/4	0	21/21	7/7	1/1	82/82	74/74	6/6
Present height (SDS) median (minimum to maximum)^*b,c,d*^	–4.01 (–14.0 to –2.08)			–4.04 (–7.30 to –2.76)			–3.48 (–7.81 to –2.00)			–3.27 (–6.95 to –2.03)		
Present height (SDS) median (minimum to maximum)^*b*,*c*,*d*^	–4.71 (–14.0 to –2.08)	–3.80 (–6.45 to –2.38)	–	–3.94 (–7.30 to –2.76)	–4.54 (–5.86 to –4.02)	–	–3.48 (–7.81 to –2.00)	–3.20 (–4.79 to –2.55)	–4.69	–3.65 (–6.95 to –2.04)	–3.21 (–5.95 to –2.03)	–2.71 (–4.92 to –2.35)
Relative macrocephaly at birth^*a,e*^	41/46 (89.1%)			9/10 (90.0%)			19/28 (67.9%)			89/159 (56.0%)		
	35/37	6/9	0	6/6	3/4	0	15/21	4/7	–	68/81	21/72	0/6
Protruding forehead	29/47 (61.7%)			5/11 (45.5%)			19/29 (65.5%)			52/154 (33.8%)		
	29/38	0/9	0	5/7	0/4	0	19/21	0/7	0/1	46/77	6/71	0/6
Body asymmetry	23/47 (48.9%)			1/11 (9.1%)			8/29 (27.6%)			29/160 (18.1%)		
	22/38	1/9	0	1/7	0/4	0	8/21	0/7	0/1	26/82	3/72	0/6
Feeding difficulties and/or low BMI^*b*^	28/46 (60.9%)			4/11 (36.4%)			21/29 (72.4%)			77/160 (48.1%)		
	27/38	1/8	0	4/7	0/4	0	19/21	1/7	1/1	64/81	12/73	1/6
Triangular face	42/47 (89.4%)			7/10 (70.0%)			18/29 (62.1%)			71/148 (48.0%)		
	34/38	8/9	0	3/6	4/4	0	15/21	3/7	0/1	46/73	24/69	1/6
Fifth finger clinodactyly	33/46 (71.7%)			2/10 (20.0%)			13/29 (44.8%)			46/147 (31.3%)		
	30/38	3/8	0	1/6	1/4	0	11/21	2/7	0/1	33/72	13/69	0/6
Muscular hypotonia	8/42 (19.0%)			2/10 (20.0%)			12/28 (42.9%)			11/114 (9.6%)		
	8/36	0/6	0	1/6	1/4	0	10/20	1/7	1/1	10/53	1/55	0/6
Speech delay	8/34 (23.5%)			4/10 (40.0%)			17/29 (58.6%)			30/106 (28.3%)		
	8/29	0/5	0	2/6	2/4	0	13/21	3/7	1/1	18/49	12/52	0/5
Congenital heart diseases	5/44 (11.4%)			0/10 (0%)			4/25 (16.0%)			25/117 (21.4%)		
	4/37	1/7	0	0/6	0/4	0	3/17	0/7	1/1	13/55	11/56	1/6
Genitourinary anomaly	12/44 (27.3%)			0/10 (0%)			7/27 (25.9%)			18/116 (15.5%)		
	11/37	1/7	0	0/6	0/4	0	4/19	2/7	1/1	14/54	4/56	0/6

Abbreviations: *H19*LOM, loss of methylation of the *H19/IGF2*:intergenic-differentially methylated region; UPD(7)mat, maternal uniparental disomy chromosome 7; IDs, imprinting disorders; NH-CSS, Netchine-Harbison clinical scoring system; SRS, Silver-Russell syndrome; SGA-SS, small-for-gestational-age with short stature; BW, birth weight; BL, birth length; SDS, standard deviation score; BOFC, birth.^*a*^Birth weight, length, and occipitofrontal circumference at birth were evaluated by sex-matched and gestational-age–matched Japanese reference data (http://jspe.umin.jp/medical/chart_dl.html).^*b*^Postnatal height, BMI, and weight were evaluated by sex-matched and age-matched Japanese reference data (http://jspe.umin.jp/medical/chart_dl.html).

^*c*^If we did not get information at 24 ± 1 months, we used the data at the nearest measure available older than 25 months before initiating growth hormone treatment.

^*d*^Height at 24 ± 1 months ≤ –2 SDS or height ≤ –2 SDS below mid-parental target height. Mid-parental target height was calculated as follows: ((father’s height + mother’s height)/2)) + 6.5 cm for boys and – 6.5

^*e*^Head circumference at birth ≥ 1.5 SDS above birth length and/or weight SDS.

All 3 patients with MLID were classified into the SRS-compatible group. Thus, the frequency of MLID was 2.0% of patients in the SRS-compatible group and 4.5% of patients with various IDs in the SRS-compatible group. MLID 1 had 5 NH-CSS items other than body asymmetry, and also showed triangular face, fifth finger clinodactyly, cryptorchidism, hypospadias, and pelvic bone defect. His BW, BL, and present height were −4.2, −3.8, and −5.8 SDS, respectively. MLID 2 had 5 NH-CSS items other than feeding difficulties together with fifth finger clinodactyly. Her BW, BL, and present height were −3.5, −3.4, and −3.3 SDS, respectively. Clinical features of MLID 3 have been reported previously (18). In brief, she showed 6 NH-CSS items and developmental delay. Her BW, BL, and present height were −3.8, −2.9, and −4.4 SDS, respectively.

#### Clinical features of patients with IDs other than *H19*LOM and UPD(7)mat

We summarized clinical features of 29 patients with IDs other than *H19*LOM and UPD(7)mat in Table 2. Of 29 patients, 21 patients were classified into the SRS-compatible group, 7 patients were classified into the non-SRS group, and 1 patient was classified into the non-SRS with microcephaly. BOFC was relatively preserved in patients with UPD(16)mat and lower in patients with PWS than those with other etiologies. TS14, which was most frequently detected in IDs other than *H19*LOM and UPD(7)mat, was diagnosed in 6 patients with maternal uniparental disomy chromosome 14 and 5 patients with epimutation at the 14q32.2 imprinted region. Eight patients with TS14 were classified into the SRS-compatible group. The etiology with the second largest number of patients was UPD(20)mat. All patients with UPD(20)mat were classified into the SRS-compatible group and the non-SRS group. UPD(6)mat, PWS, and UPD(16)mat were detected in several patients. In addition to chromosomal abnormalities involving the imprinted region, parthenogenesis was identified in a single patient. Muscular hypotonia was observed in all patients with PWS, and about half of patients with TS14, UPD(20)mat, 11p15 maternal duplication, and UPD(16)mat. Speech delay was observed in all patients with PWS and 11p15 maternal duplication, and in about half of patients with TS14, UPD(20)mat, and UPD(16)mat. Genitourinary anomalies were frequently identified in UPD(6)mat, PWS, and UPD(16)mat.

## Discussion

In this study, to clarify the contribution of IDs to SGA-SS and the molecular and phenotypic spectrum of SRS, we performed methylation analysis for the DMRs related to known IDs in 249 patients with SGA-SS, including 92 patients referred to us for investigation for SGA-SS and 157 patients referred to us for genetic testing for SRS who were classified into 3 subgroups based on NH-CSS. A total of 30.4% of patients in the SRS-compatible group, 13.8% in the non-SRS group, and none of patients in the non-SRS with microcephaly group had major genetic causes of SRS such as *H19*LOM and UPD(7)mat. These results suggest that *H19*LOM and UPD(7)mat do not contribute to the development of SGA-SS with relative microcephaly. The frequency of *H19*LOM and UPD(7)mat in the SRS-compatible group in our study was lower than those (76.7%) in a previous study, which examined 60 patients with 4 or more NH-CSS items (12). Interestingly, IDs other than *H19*LOM and UPD(7)mat were detected in 14.2% of patients in the SRS-compatible group, in 7.4% of patients in the non-SRS group, and 14.3% of patients in the non-SRS with microcephaly group. Particularly, TS14 and UPD(20)mat were detected in at least 5 patients. A previous study reported the results of methylation analysis for 11 imprinted loci (*PLAGL1*, *IGF2R*, *PEG10*, *MEST1*, *GRB10*, *KCNQ1OT1*, *H19*, *IGF2P0*, *DLK1*, *PEG3*, and *NESPAS*) in 79 patients with growth restriction who were classified into 4 clinical referral categories, namely, SRS, intrauterine growth restriction (IUGR), short stature (SS), and IUGR and SS (15). In this study, 21 of 34 patients with SRS and 1 of 12 patients with IUGR and SS had *H19*LOM; however, there were no patients with other etiologies other than *H19*LOM related to known IDs. In addition, this study did not perform clinical assessment of these patients based on clinical diagnostic criteria of SRS. Our study examined all the known IDs in patients with SRS and/or SGA-SS. Our study reveals the contribution of various IDs for SGA-SS.

Clinical analysis among patients with *H19*LOM, UPD(7)mat, IDs other than *H19*LOM and UPD(7)mat, and unknown etiology produced several notable clinical findings. First, 21 patients satisfying NH-CSS had etiologies other than *H19*LOM and UPD(7)mat, and meanwhile, 13 patients with *H19*LOM and UPD(7)mat did not satisfy NH-CSS. Consistent with this, Azzi et al reported a single patient with UPD(7)mat who did not satisfy NH-CSS (12), and Eggermann et al reported that *H19*LOM and UPD(7)mat carriers do not always show the unambiguous SRS phenotype (21). The diagnosis of SRS based on NH-CSS items consisting of nonspecific clinical features may result in a wide molecular spectrum of SRS. With regard to patients with *H19*LOM and UPD(7)mat having SGA-SS without typical SRS phenotype, some other factors such as environmental, maternal, paternal, placental, and other genetic factors may affect their phenotypes. Second, most patients with *H19*LOM, two-thirds of patients with UPD(7)mat, and two-thirds of patients patients with IDs other than *H19*LOM and UPD(7)mat satisfied NH-CSS. Patients with *H19*LOM showed the most severe prenatal and postnatal growth failure. In addition, relative macrocephaly, protruding forehead, and body asymmetry were frequently detected in patients with *H19*LOM. Patients with UPD(7)mat had heavier BW and taller BL than patients with *H19*LOM; however, patients with UPD(7)mat showed severe postnatal growth failure as well as those with *H19*LOM. Furthermore, 90% of patients with UPD(7)mat had relative macrocephaly, whereas only 9% had body asymmetry. These results indicate the differences in clinical features related to NH-CSS between patients with *H19*LOM and patients with UPD(7)mat, as previously reported (7, 12). Third, regarding clinical features other than NH-CSS items, muscular hypotonia was found in about 40% of patients with IDs other than *H19*LOM and UPD(7)mat. This is probably because patients with TS14 and PWS, who have muscular hypotonia with high frequency (8, 9), occupied half of the patients with IDs other than *H19*LOM and UPD(7)mat. Speech delay was observed in higher frequency in patients with UPD(7)mat, and those with IDs other than *H19*LOM and UPD(7)mat, than in those with *H19*LOM. In this regard, abnormal gene expression of *FOXP2* on chromosome 7 associated with language development and dysfunction of the genes related to language development caused by IDs may lead to speech delay (22, 23). CHDs were detected in 16% of IDs other than *H19*LOM and UPD(7)mat. Previous studies also showed that some DNA methylation abnormalities were associated with CHD (24, 25). For SGA-SS patients with CHD, we need to consider the examination of IDs other than *H19*LOM and UPD(7)mat. Genitourinary anomalies were observed in about 30% of patients with *H19*LOM. Several studies have reported that male genitourinary anomalies were frequently identified in SRS (13, 26, 27), and lower birth weight was associated with urogenital anomalies (28). However, it remains to be clarified whether SGA caused by SRS or genetic causes of SRS result in male genitourinary anomalies; our result shows the possibility that *H19*LOM itself leads to male genitourinary anomalies.

Comprehensive methylation analysis for DMRs identified patients with various IDs. Patients with UPD(11)mat mosaic or 11p15 maternal duplication showed SRS phenotype as well as previous reports (29-31). UPD(11)mat mosaic results in decreased gene expression of *IGF2* that functions as a growth promoter, increased gene expression of *CDKN1C* that functions as a negative regulator of cell proliferation (32, 33), and 11p15 maternal duplication that results in increased *CDKN1C* gene expression (33). Consistent with this, gain-of-function gene mutation in *CDKN1C* was reported in patients with SRS (34). Over 70% of patients with TS14, UPD(20)mat, and UPD(16)mat were classified into the SRS-compatible group, as well as in the previous report (35). On the other hand, 1 out of 3 patients with UPD(6)mat in our study met NH-CSS criteria. UPD(6)mat is a rare ID with only 29 cases reported (http://molbiol.sci.am/ssmc/ssmc-tl.com/upd.html, accessed Jan 1, 2020). IUGR has been reported as one of the most common features of UPD(6)mat; however, it is unknown whether SS and the remaining NH-CSS items are common features or not due to lack of clinical information about patients with UPD(6)mat (36). In our study, speech delay was not observed, but genitourinary anomalies were detected in 2 patients with UPD(6)mat. Consistent with this, previous studies reported some patients having abnormal genitalia and mild speech delay (37, 38). Further studies are required to clarify the clinical features of patients with UPD(6)mat.

In our study, the frequency of MLID was 3.4% in the patients with various IDs, and 4.5% in patients with various IDs classified into the SRS-compatible group. Recently, Eggermann et al reported that the frequency of MLID was 3.6% in SRS patients with aberrant findings (39). These findings suggest that MLID in patients with SRS is less frequent. All patients with MLID in our study fulfilled NH-CSS criteria (5-6 out of 6 items) and showed prenatal and postnatal growth failure. Only a single patient had additional congenital abnormalities, namely, cryptorchidism, hypospadias, and pelvic bone defect. Although Pooles et al reported that patients with MLID more frequently exhibit growth delay and additional congenital abnormalities (40), other reports showed that there were no significant differences in the clinical findings between SRS patients with MLID and “isolated” SRS patients (41, 42). Our results were partially consistent with previous reports, specifically, severe postnatal growth failure in 2 patients. To clarify the clinical features of the patients with MLID, further accumulation of cases with MLID are required.

This study has some limitations. First, because our laboratory is one of the largest facilities that can perform genetic analyses of SRS in Japan, some selection bias for recruiting patients may occur, that is, patients with SGA-SS clinically diagnosed by attending physicians have some aspect of SRS phenotype. Second, many patients included in our study were preschool children under 3 years old. It is possible that developmental delay and characteristic features of each etiology were unclear at the time of examination. Lastly, we did not perform mutation screening of genes that cause genetic syndromes leading to SGA-SS. Patients with unknown etiology in our study may have had gene mutations of genetic syndromes resulting in SGA-SS.

In conclusion, we clarified the contribution of IDs to SGA-SS. This study also showed broad molecular and phenotypic spectrums of SRS. Various IDs constitute underlying factors for SGA-SS, including SRS.

## Data Availability

Some or all datasets generated during and/or analyzed during the current study are not publicly available but are available from the corresponding author on reasonable request.

## Abbreviations

aCGH: array comparative genomic hybridization
BL: birth length
BMI: body mass index
BOFC: birth occipitofrontal circumference
BW: birth weight
CHD: congenital heart disease
CNV: copy number variant
DMR: differentially methylated region
ID: imprinting disorder
IUGR: intrauterine growth restriction
MLID: multilocus imprinting disturbance
NH-CSS: Netchine-Harbison clinical scoring system
PCNV: pathogenic copy number variant
PWS: Prader-Willi syndrome
SDS: standard deviation score
SGA: small for gestational age
SGA-SS: small-for-gestational-age with short stature
SRS: Silver-Russell syndrome
SS: short stature
TS14: Temple syndrome
UPD(7)mat: maternal uniparental disomy chromosome 7
UPD(16)mat: maternal uniparental disomy chromosome 16
UPD(20)mat: maternal uniparental disomy chromosome 20

## Additional Information

***Disclosure Summary:*** The authors report no conflicts of interest.
